# Multimodality Imaging in the Diagnosis of an Early Tako-Tsubo Syndrome Recurrence

**DOI:** 10.3390/diagnostics16020292

**Published:** 2026-01-16

**Authors:** Maria Letizia Berloni, Andrea Daniele Annoni, Marco Moltrasio, Andrea Baggiano, Gianluca Pontone

**Affiliations:** 1 Cardiology Unit, University of Ferrara, 44124 Ferrara, Italy; 2 Department of Perioperative Cardiology and Cardiovascular Imaging, Centro Cardiologico Monzino IRCCS, 20138 Milan, Italy; 3 Department of Emergency-Urgency, Centro Cardiologico Monzino IRCCS, 20138 Milan, Italy; 4 Department of Biomedical, Surgical and Dental Sciences, University of Milan, 20122 Milan, Italy

**Keywords:** Tako-Tsubo syndrome, cardiac CT, cardiac magnetic resonance, multimodality imaging

## Abstract

We report the case of an 80 yo female patient with cardiovascular risk factors and previous diagnosis of Tako-Tsubo syndrome, who was referred to our institution one year after a previous diagnosis, due to symptoms suggestive of acute coronary syndrome (SCA) after severe emotional stress. After ruling out suspected CAD by cardiac computed tomography (CCT) and subsequent invasive coronary angiography (ICA) confirming no significant stenosis but presence of vulnerable plaque, the patient underwent further investigation by cardiac magnetic resonance (CMR) that confirmed a clinical picture compatible with recurrence of Tako-Tsubo syndrome. Our case underlines the importance of multimodality imaging to guide diagnosis and treatment in this specific clinical scenario.

**Figure 1:**
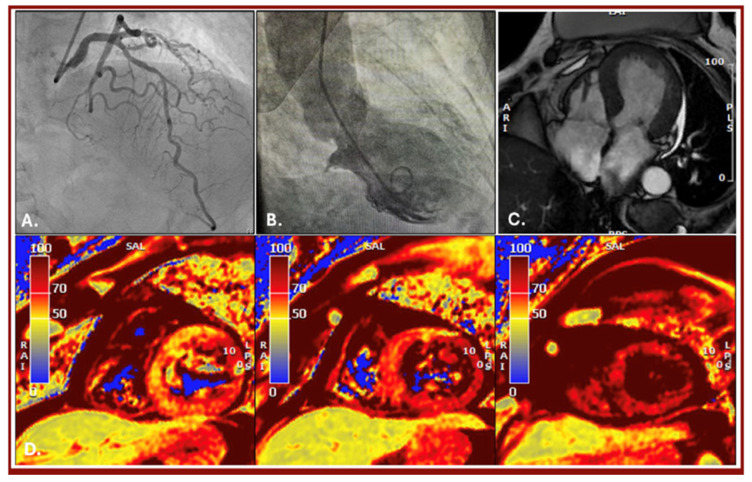
Panels relating to first hospital admission in 2022. (**A**): Coronary angiography showing no significant coronary artery disease (CAD). (**B**): Intraprocedural ventriculography demonstrating hyperkinesis of the basal segment and akinesia of the mid-apical segment. (**C**): Long-axis steady-state free precession (SSFP) cardiac magnetic resonance (CMR) showing apical LV dilation. (**D**): High T2 signal short-axis images (CMR) of basal segments, mid segments and apical segments, respectively (T2 mean values of 68, 76 and 79 ms in basal, mid and apical segments, respectively). We report the case of an 80-year-old female patient with a history of hypertension, type 2 diabetes mellitus complicated by peripheral neuropathy, and a previous laparoscopic hysterectomy for uterine fibromatosis. In late 2022, the patient was admitted to our emergency department for chest pain and ECG abnormalities, (lateral nonspecific ST-T wave abnormalities), and echocardiography revealed a dilated left ventricle with apical akinesia and a moderate reduction in systolic function (LVEF 40%). Due to suspected coronary artery disease (CAD), a coronary angiography was performed, without evidence of significant CAD (**A**). Additionally, an intraprocedural ventriculography showed LV dilation with akinesia of the mid-apical segments and compensatory hyperkinesis of the basal segments (**B**). Due to these findings, and based on suspicion of Tako-Tsubo syndrome, the patient underwent cardiac magnetic resonance (CMR), which confirmed the findings, showing LV apical segment dilation (**C**) with diffuse edema on T2 mapping (**D**) without ischemic features and moderate left ventricle systolic function. The patient was discharged after appropriate treatment, with a diagnosis of Tako-Tsubo syndrome and residual moderate LV ejection fraction reduction. At a subsequent follow-up visit three months after discharge, the patient showed regression of symptoms with recovery of left ventricle systolic function on echocardiogram (LVEF 56%).

**Figure 2:**
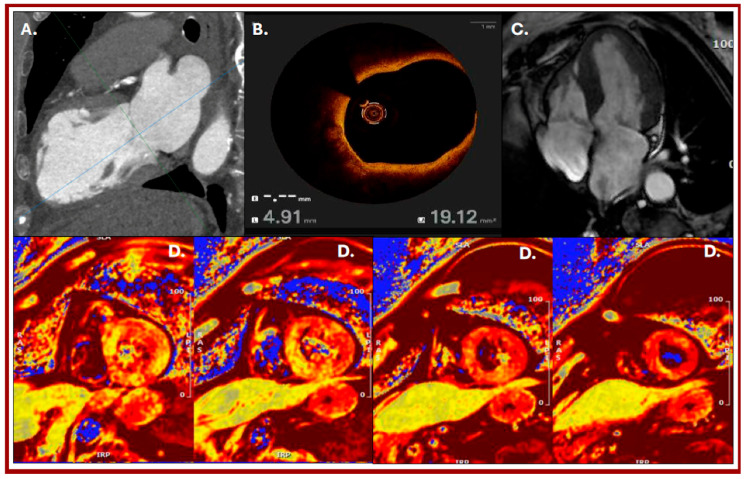
Panels relating to second hospital admission in 2024: (**A**): Left ventricular CT scan, with two-chambers view, showing dilation of the mid-apical segments. (**B**): Intravascular imaging of vulnerable plaque (OCT) conducted during ICA. (**C**): Long-axis SSFP CMR imaging confirming apical LV dilation. (**D**): High T2 signal in short-axis images of the left ventricle due to diffuse edema of the left ventricle (T2 mean values of 65, 78 and 82 ms for basal, mid and apical segments, respectively). In early 2024, the patient was admitted to another center for laparoscopic surgical lysis of an adhesive band. After discharge, she reported intense chest pain, accompanied by anxiety related to the postoperative period. Thus the patient was referred again to our emergency department. Initial evaluation revealed a peak high-sensitivity troponin I (TnIhs) level of 1846 ng/L, without ECG abnormalities, but echocardiography showed apical akinesia of left ventricle with moderate LVEF reduction (40–41%). The patient was subsequently hospitalized, and coronary CT angiography (CCTA) revealed dilation of the mid-apical segments of the LV (**A**) and insignificant CAD. Due to her history, the patient underwent coronary angiography that confirmed insignificant stenosis of the mid-left LAD with evidence of vulnerable plaque on intravascular perioperative imaging (**B**). Despite the presence of coronary artery disease, due to patient history the suspicion of Tako-Tsubo syndrome recurrence remained, and the patient was referred for further examinations: a CMR was performed, confirming apical ballooning with moderate global systolic function reduction and LV apical segment edema (**C**). Specific T2 mapping CMR sequences showed concurrent edema of the LV especially in the mid-apical segments (**D**). During the hospitalization, the ECG evolved, showing the onset of negative T-waves in the anterolateral leads. After appropriate treatment the patient was discharged with diagnosis of Tako-Tsubo syndrome recurrence. At six months follow-up ecochardiography showed left ventricle function recovery (LVEF 57%). Tako-Tsubo syndrome is an acute cardiac syndrome that typically presents with abnormalities in segmental kinetics, leading to acute heart failure in the absence of a culprit epicardial coronary artery disease [1] and is associated with a very low recurrence rate, estimated at 1.5% [2]: some studies have observed a recurrence rate up to five times higher in young women and patients with greater left ventricular dysfunction, suggesting a higher susceptibility to stress-related events. However, these factors are still under investigation, as is the use of appropriate therapies for recurrence prevention. Beta-blockers are commonly used empirically to prevent Tako-Tsubo recurrences, as are angiotensin-converting enzyme (ACE) inhibitors and angiotensin receptor blockers, although there is no strong evidence supporting their effectiveness [3,4]. Data concerning the recurrence rate of Tako-Tsubo syndrome, patient clinical characteristics, and outcomes have also been collected in the multicenter GEIST registry, including data from nine European centers, providing a broader perspective on this cardiomyopathy, showing how the estimated recurrence rate remains very low (approximately 4%) with most recurrences occurring within five years after the initial event [5]. Our case shows how multimodality imaging is crucial for an accurate diagnosis in this setting. The diagnosis of Tako-Tsubo syndrome, including its recurrence, initially requires the exclusion of CAD and in this scenario CCT is the first-line non-invasive imaging modality of choice. Furthermore, among non-invasive imaging, CMR has a pivotal role in its definitive diagnosis, allowing kinesis defects and tissue characterization. Thus multimodal imaging offers an added value compared to individual techniques, enabling faster and more accurate diagnosis of Tako-Tsubo syndrome and its recurrences, enhancing the downstream management of these patients with significant implications for daily clinical practice.

## Data Availability

The data presented in this study are available upon request from the corresponding author due to privacy reasons.

## Abbreviations

The following abbreviations are used in this manuscript:

ACE	Angiotensin-Converting Enzyme
ACS	Acute Coronary Syndrome
CAD	Coronary Artery Disease
CCT	Cardiac Computed Tomography
CCTA	Coronary Computed Tomography Angiography
CMR	Cardiac Magnetic Resonance
ECG	Electrocardiogram
ICA	Invasive Coronary Angiography
LAD	Left Anterior Descending (coronary artery)
LV	Left Ventricle
OCT	Optical Coherence Tomography
PCI	Percutaneous Coronary Intervention
Hs-cTnI	High-Sensitivity Troponin I
